# Involution Melancholia*Read before the Fourth District Medical Society, San Angelo, Texas, November 2, 1913.

**Published:** 1916-02

**Authors:** J. A. McIntosh

**Affiliations:** San Antonio, Texas


### TEXAS MEDICAL JOURNAL

Dr. F. E. Daniel, Founder. Established July, 1885

MRS. F. E. DANIEL, - Publisher and Managing Editor

Published Monthly.—Subscription, $1.00 a Year.

VOL. XXXI AUSTIN, FEBRUARY, 1916 No. 8

The publisher is not responsible for views of contributors

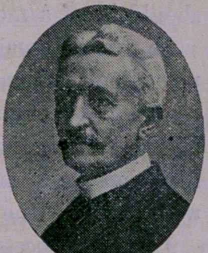

### Original Articles.

### Involution Melancholia.*

BY J. A. M'INTOSH, M. D., SAN ANTONIO, TEXAS.

In contrast to the phychoses of adolescence, the time when the individual is undergoing evolution or experiencing those changes incident to the passing from the stage of childhood into that of an adult, I wish to call your attention to a mental disturbance met with in another period of life in which there may be just as serious consequences as the result of certain retrograde changes occurring during the process of involution.

This disease is met with in both men and women, but more frequently in the latter. It develops usually between the ages of 40 and 50 years in women; in other words, at the time of the menopause, but about ten years later in men. At this time of life both men and women have visions of unattained ambitions and realize that the opportunity for attaining them is gone. Ideals which have been moral, mental and physical stimuli have been uprooted and there is left a soil fertile for the growth of pessimism and despondency. To a woman especially, the awakening to oncoming old age with what so many believe to be unattractiveness is a burden to some. This age, too, finds many unprovided for, and this is especially trying on the man who has been active and able to provide for himself and family, but now finds that on account of a depreciation in his physical and mental vitality he is more or less dependent. It is a small wonder, then, that this period of life should not only contribute a large proportion

*Read before the Fourth District Medical Society, San Angelo, Texas, November 2, 1913.

to the ranks of nervous invalids, but that the type of trouble should be of a melancholy nature.

In the language of Dr. Peterson, of New York, "There is a physiological commotion in the nervous system at the time of the cessation of ovulation and menstruation, a disequilibration associated even in normal individuals with numerous neurotic manifestations, and in such as have unstable organizations, attended with peril to the mental integrity."

In this process of involution there are retrograde changes in most of the organs of the body, but especially in the generative ones. The destructive changes are in excess of the building up processes, and consequently the whole system is more or less under the influence of these destroyed tissues in the process of absorption and elimination. This substance acts as a toxine, and the process may be very fittingly described as one of auto-intoxication. And, too, there is an absence or at least a lessened amount of the internal secretions from the various atrophied and nonfunctioning organs. As these secretions are now believed to play a very important rôle in the maintenance of good health, so their absence is to be considered as, at least, a possible factor in the etiology of the disease under consideration.

The symptoms of this disease are not different from those of other depressive insanities, some authors classing it as one form of manic-depressive insanity, and I deem it unnecessary to go into a detailed description of its various manifestations. Profound depression, intense anxiety and delusions are the prominent symptoms. In all of these cases there is a strong suicidal tendency, and refusal of food is nearly always present. Orientation and memory are, as a rule, undisturbed. The delusions are usually of sin, or unpardonable sin, and the patient is in constant fear of being destroyed, most commonly by fire.

The course of this disease is, because of its etiology, a comparatively long one, but as in other diseases varies somewhat in different individuals. Of the 1541 patients that have been under my observation in Dr. Moody's sanitarium the past eight years, 106 or 7 per cent have been cases of involution melancholia; 56 of this number were women and their average age was 47 years. The average age for the men was 55 years. Of this 106 cases treated 39 were discharged unimproved. This 39 includes four deaths which occurred soon after admission, and the average length of stay under treatment by the other 35 was less than one month. Only four of this number were treated for more than three months, and none for more than six months. Of those who recovered or were discharged much improved, 67 in number, the average length of time under treatment was, for the women, eleven months, and, for the men, five months. We can readily see from these figures that the duration in men is much shorter than in women, and it has been my observation that the percentage of recoveries is greater in the former, and I believe it would be still greater could the men be persuaded to stay away from business and take treatment long enough. But we find it much more difficult to keep a man under treatment running over a period of several months than a woman, because in the case of a man who is the bread-earner and supporter of the family there is not only the actual cost of the treatment to be considered but in many cases his salary stops and his business suffers because of his absence. For these reasons most men insist on going back to their business before they are entirely well and thus run the risk of a return of the trouble or a lack of complete recovery. In many of our cases, even after eight or ten months' treatment, we have been unable to see enough change in the patient's symptoms to give a very favorable prognosis, and still the patient recovers completely in two or three more months. I believe very firmly that there are many unfortunate patients who have gone on in mental unsoundness through their remaining years for no other reason than that their relatives and their doctor have become discouraged and given up all hope after a few months treatment, without recovery. Consequently, this individual must not only be deprived of all future happy associations with his family and friends but must help to fill the already crowded asylums.

To emphasize the importance of continued treatment through many months duration, if necessary, I wish to cite the following cases:

Case No. 1.—Mrs. F., admitted May 27, 1908, age 57. Family history good. Nothing of special importance in personal history except that she had a severe spell of la grippe two years previously and had menstruated last at the age of 50 years. For several months before admission she had been sleeping poorly and slowly showing signs of despondency. For several months while under treatment she would not talk, and ate very poorly. In fact, for several months at a time it was necessary to use forced feeding. Many times her husband became very much discouraged, and it was by repeatedly telling him that his wife was suffering from a long-coursed disease and that there was still a chance for her recovery that he kept up the treatment. These symptoms continued, sometimes ameliorating and then worse again, until about six weeks before she was discharged. From this time on she would talk, take interest in her surroundings and inquire of relatives, asking how long before she could go home, etc. She was discharged restored May 2, 1909 after eleven and one half months' treatment, and has been at home in good health since, notwithstanding the death of a son and her husband since leaving the institution.

Case No. 2.—Mrs. A., admitted November 3, 1912, age 35. Bad heredity. Hair extremely gray. Menstruation present but scanty. Nothing different in symptoms except that patient believed she was going to be burned alive, and often said she could hear them preparing the furnace for her cremation. These symptoms appeared four or five months before admission and continued unchanged for more than a year after admission. In January, 1914, these symptoms began to ameliorate, and patient was not so persistent in her perverted ideas; in fact, she could be persuaded for the time being that she was wrong. This improvement continued and patient was discharged, recovered, March 21, 1914, after sixteen and one-half months continuous treatment. Several times during this period her husband, on account of shortness of funds, almost decided to remove her to the asylum, but refrained, as we told him there was still hope of recovery.

Case No. 3.—Female, age 53, with bad heredity, recovered after five years' continuous treatment.

I have cited these few cases to emphasize the importance of not giving a too unfavorable prognosis in these cases even after several months duration, especially if proper treatment can be carried out. Most authors place the percentage of recoveries in this disease at 35 per cent and less. This is based mainly on the reports from the State institutions where the patient, as a rule, is not taken for treatment until the disease has continued for some time, and many times is classed with the chronic insane from the time of admission. We have learned that many of our patients who were discharged improved after a few months treatment have continued this improvement to a complete recovery. Consequently, the statistics I have given you will justify a recovery percentage of 55 per cent or 60 per cent, considering all cases admitted. Should we consider only those who remained under treatment longer than six weeks, even the percentage would be very materially increased.

The treatment of this disease does not require the use of many drugs. In fact, drugs without proper management in other particulars are of little of no avail. I consider location of the patient in a well-regulated institution properly equipped for the care and treatment of mental diseases of supreme importance, and should be obtained before any other treatment is begun. To quote the celebrated alienist, our late Dr. S. Wier Mitchell, of Philadelphia: "It is rare to find any nervous patient so free from the influence of their habitual surroundings as to make it easy to treat them in their own homes. It is needful to disentangle them from the meshes of old habits and to remove them from contact with those who have been the willing slaves of their caprices. Once separate the patient from the moral and physical surroundings which have become a part of her life of sickness, and you will have made a change which will be in itself beneficial and will enormously aid in the treatment which is to follow." Constant supervision to protect our patients from suicide is necessary, and the patient must be given nourishment despite her refusal of food. To prevent exhaustion, especially in the anxious cases, often requires much thought and very careful nursing. Plenty of rest (including sleep) and nourishment are absolutely necessary with each patient, and the case will terminate in a very short time if these two requirements are not met. For the relief of the insomnia hydrotherapy or some other means than resort to drug hypnotics is preferable. In many cases, however, I find it necessary to use some trional and veronal. There is no specific remedy in the way of drugs to be given these cases; nerve tonics and nerve alteratives are of value, and should be given, selecting the ones most suitable for the individual case after considering carefully all the physical needs of the patient. Tonics of varying combinations and doses, of iron, strychnine and arsenic will usually meet all the requirements along this line.

From my brief outline of the indications to be met in the treatment of this disease, you can readily see how difficult it would be to treat a case under home surroundings, especially over a period of several months. I believe the percentage of recoveries will increase as the laity as well as the members of our own profession know more about this disease and its requirements, and realize the importance of placing the patient under proper treatment, and that, too, at the time of the onset of the disease.

The public knows a great deal about the various physical diseases and insist on certain treatments being carried out for their relief. For instance, when you tell a mother her child has diphtheria, she immediately wants him to have the antitoxine, and will get another doctor and probably sue you for malpractice if you fail to give it. If you make a diagnosis of typhoid fever, every one says the patient must have only liquid diet, and so with many diseases, and the number is increasing as the public becomes familiar with the indications to be met. This same demand for proper treatment of the various psychoses and neuroses should exist and will just as soon as our patrons know more about these diseases, and I believe it our duty to educate the people along this line. It will make it easier for the doctor to carry out the proper line of treatment in each case, as the patient's relatives will be not only willing but anxious to co-operate in a every detail of treatment and urge its early beginning, and, best of all, the percentage of recoveries will be much increased, and thus many who are otherwise doomed to a life of mental invalidism and added to the long list of dependents will be restored to good health and a life of happiness with those whom they love.

